# Elastase treatment of tendon specifically impacts the mechanical properties of the interfascicular matrix

**DOI:** 10.1016/j.actbio.2021.01.030

**Published:** 2021-03-15

**Authors:** Marta S. Godinho, Chavaunne T. Thorpe, Steve E. Greenwald, Hazel R.C. Screen

**Affiliations:** a Institute of Bioengineering, School of Engineering and Materials Science, Queen Mary University of London, London, E1 4NS, United Kingdom; b Comparative Biomedical Sciences, The Royal Veterinary College, Royal College Street, London, NW1 0TU, United Kingdom; c Blizard Institute, Barts and London School of Medicine and Dentistry, Turner Street, London E1 11BB, United Kingdom

**Keywords:** Tendon, Elastin, Elastase, Interfascicular matrix, Fatigue

## Abstract

The tendon interfascicular matrix (IFM) binds tendon fascicles together. As a result of its low stiffness behaviour under small loads, it enables non-uniform loading and increased overall extensibility of tendon by facilitating fascicle sliding. This function is particularly important in energy storing tendons, with previous studies demonstrating enhanced extensibility, recovery and fatigue resistance in the IFM of energy storing compared to positional tendons. However, the compositional specialisations within the IFM that confer this behaviour remain to be elucidated. It is well established that the IFM is rich in elastin, therefore we sought to test the hypothesis that elastin depletion (following elastase treatment) will significantly impact IFM, but not fascicle, mechanical properties, reducing IFM resilience in all samples, but to a greater extent in younger tendons, which have a higher elastin content. Using a combination of quasi-static and fatigue testing, and optical imaging, we confirmed our hypothesis, demonstrating that elastin depletion resulted in significant decreases in IFM viscoelasticity, fatigue resistance and recoverability compared to untreated samples, with no significant changes to fascicle mechanics. Ageing had little effect on fascicle or IFM response to elastase treatment.

This study offers a first insight into the functional importance of elastin in regional specific tendon mechanics. It highlights the important contribution of elastin to IFM mechanical properties, demonstrating that maintenance of a functional elastin network within the IFM is essential to maintain IFM and thus tendon integrity.

**Statement of significance:**

Developing effective treatments or preventative measures for musculoskeletal tissue injuries necessitates the understanding of healthy tissue function and mechanics. By establishing the contribution of specific proteins to tissue mechanical behaviour, key targets for therapeutics can be identified. Tendon injury is increasingly prevalent and **c**hronically debilitating, with no effective treatments available.

Here, we investigate how elastin modulates tendon mechanical behaviour, using enzymatic digestion combined with local mechanical characterisation, and demonstrate for the first time that removing elastin from tendon affects the mechanical properties of the interfascicular matrix specifically, resulting in decreased recoverability and fatigue resistance. These findings provide a new level of insight into tendon hierarchical mechanics, important for directing development of novel therapeutics for tendon injury.

## Graphical abstract

**Figure:**
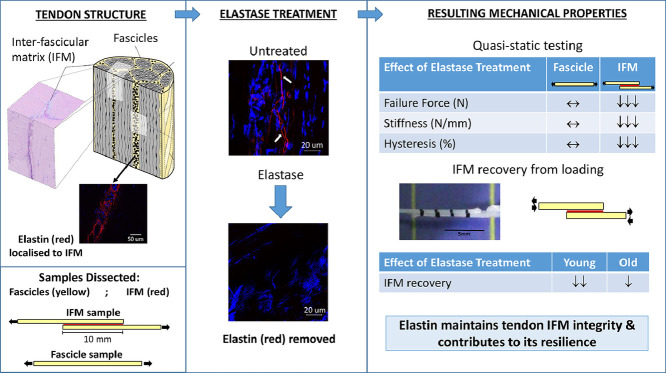

## 1 Introduction

Tendons possess a highly organised fibre composite structure, in which the primarily collagenous subunits are surrounded by a softer matrix material. At the highest structural level, the collagen-rich fascicles are bound together by a proteoglycan- and elastin-rich interfascicular matrix (IFM, also referred to as endotenon) [1], [2].

Whilst all tendons transfer force from muscle to bone, the stresses and strains to which different tendons are subjected vary considerably, and many highly loaded tendons additionally act as energy stores, with increased extensibility and recoverability to improve locomotion efficiency [3], [4]. Previous studies have demonstrated that the specialised mechanical properties of energy storing tendons such as the equine superficial digital flexor tendon (SDFT), primarily result from compositional and mechanical specialisation in the IFM [5], [6], [7]. The IFM enables non-uniform loading and increased overall extensibility of tendon as a result of low stiffness behaviour under small loads, which enables fascicle sliding. The capacity for fascicle sliding has been shown to be significantly greater in energy storing tendons, likely enabled by enrichment of the energy storing tendon IFM with elastin and lubricin [8], [9].

With further studies demonstrating that IFM extensibility and fatigue resistance both reduce with ageing [10], [11], it has been hypothesised that the increased injury risk seen in aged energy storing tendons originates from ageing changes in the IFM, and that poor IFM specialisation may be a primary cause of tendon overload damage. Unravelling the mechanisms that facilitate energy storage and their impact on injury risk has exciting implications for treating tendon injuries, not only providing the functional understanding from which to develop treatments, but also for targeting preventive approaches, such as mitigating the age-related loss of tendon resilience.

Recent studies have shown that, while the IFM is composed of a variety of collagens, predominantly types I and III, it is also rich in proteoglycans, particularly lubricin, and is a highly cellular region of the tendon [7], [8]. Studies on a range of murine, equine, human and bovine tissues have indicated that elastin makes up only 2–5% of the dry weight of tendon [9], [12], [13], [14]. However, studies in large animal or human tendons have consistently shown that it is predominantly localised to the IFM (~90%), with light microscopy imaging suggesting elastic fibres may bridge adjacent fascicles [8], [9]. Elastin is characterised by highly compliant and resilient behaviour, and has previously been shown to be more abundant in equine energy storing tendons (approximately 3% dry weight), compared to positional tendons (1.5%) [1], [8], [9], [15], suggesting that it may be of particular importance in tendon energy storage. Further, elastin content decreases with ageing in the energy storing SDFT, which may contribute to the increased injury risk observed with ageing specifically in energy storing tendons [9], [16], [17].

Previous studies focussed on elucidating the contribution of elastin to tendon mechanics have identified alterations in failure stress and strain [18], stiffness [19] and shear response [20] as a result of elastin depletion, utilising either heterozygous knockout models or enzymatic digestion of elastin. However, to the authors’ knowledge, no studies have investigated the effect of elastin depletion in energy storing tendons, or directly determined the effect of elastin depletion on IFM mechanics.

The current study thus focuses on a detailed exploration of the role of elastin in tendon function, carrying out a region-specific analysis of how elastin depletion impacts IFM and fascicle function in energy storing tendons, and how this is affected by ageing.

We utilise the horse superficial digital flexor tendon (SDFT) as a relevant and accepted energy storing tendon model, showing similar disease pathology and epidemiology to that seen in human energy storing tendons [17]. We combine enzymatic digestion with a series of specialised mechanical measurements to test the hypothesis that elastin depletion will significantly impact the mechanical properties of the IFM, but not those of the fascicle, reducing IFM resilience in all samples, but to a greater extent in younger tendons.

## 2 Methods

### 2.1 Sample collection and preparation

SDFTs from 5 young (3 to 7 years – young group) and 5 old (15 to 19 years - old group) horses were dissected from both forelimbs of horses euthanised at a commercial abattoir within 24 h post-mortem. Tendons were divided into four equal sized longitudinal quarters, all obtained at laterally adjacent sections of the mid-metacarpal region. Sections were wrapped in paper tissue dampened with phosphate-buffered saline (PBS) and aluminium foil, and stored at −20 °C until required, enabling the multiple quasi-static, fatigue and recovery experiments to be conducted on separate sections, allocated at random, avoiding multiple freeze-thaw cycles.

### 2.2 Optimisation and validation of elastase digestion protocol

20 samples, each composed of 2 fascicles bound together by IFM (approximately 40 mm long) were dissected from a single young SDFT section (*n* = 1; 7 years old) to optimise and validate the elastase digestion protocol. Samples were divided into 4 groups: fresh, control, 0.2 U/ml elastase and 2 U/ml elastase, with the chosen enzyme concentrations based on previous literature [21] in conjunction with a preliminary study. Samples in the “fresh” group were stored at 4 °C and analysed within 16 h of dissection, whilst samples in the two elastase groups and the control group were incubated in a buffer solution with and without the inclusion of elastase for 16 h at room temperature, with gentle agitation. The buffer solution comprised 5 ml of 1x PBS plus 0.1 mg/ml soybean trypsin inhibitor (SBTI) solution. Elastase (trypsin-free porcine pancreatic elastase, EPC134, Elastin Products Co., Owensville, MO) was added at concentrations of 0.2 U/ml or 2 U/ml, to the desired groups.

After incubation, samples were washed in PBS and divided into 2 groups for biochemical and immunohistochemical analyses. Samples for biochemical analysis were stored at −20 °C until required, while samples for immunohistochemical analysis were prepared immediately.

### 2.3 Elastin immunolocalisation

One sample from each test group was immunolabelled for elastin and cell nuclei. Samples were fixed in 4% paraformaldehyde (PFA) for 30 min, washed in PBS, then incubated in 10% Goat Serum for 1H, followed by the elastin antibody, which has previously been validated in equine tissue [9] (Ab9519; 1:100 dilution in 5% goat serum) overnight. Samples were then washed in PBS, incubated in the secondary antibody (555 Goat anti Mouse IgG *H* + *L*, 1:500 in 5% goat serum) for 1H, washed again in PBS, and finally incubated for a further 5 min in DAPI (1:1000 in 5% goat serum).

Samples were placed on poly-lysine slides, mounted with prolong Diamond antifade and imaged with a laser scanning confocal microscope (Zeiss ELYRA; Carl Zeiss AG, Oberkochen, Germany) using a 63x oil objective. Confocal z series were taken with an image size of 225 × 225 μm, pixel size of 0.11 × 0.11 μm and a z-step size of 0.25 μm.

### 2.4 Biochemical analysis

The amounts of elastin, sulphated glycosaminoglycan (GAG) and collagen type I, were quantified in fresh, control, 0.2 U/ml and 2 U/ml elastase treatment groups, combining the remaining 4 samples in each treatment group to ensure ~25 mg dry weight of tissue, on which to perform all three biochemical assays.

Samples were powdered using a Micro dismembrator, freeze dried and then weighed. Approximately 6 mg of powered sample was used to determine elastin content with the Fastin Elastin assay (Biocolor, UK). To briefly describe the procedure (fully detailed in [9]), elastin was extracted from the tendon samples using oxalic acid, and alpha-elastin was used to create a standard curve.

The remaining tissue (10–20 mg dry weight) was solubilised in papain (P3125, Sigma, UK) for 18 h at 60 °C, prior to measurement of sulphated GAG and collagen content using standard DMMB and hydroxyproline assays respectively [22], [23].

Results revealed that treatment with 2 U/ml elastase caused a 70% reduction in elastin content, and therefore this concentration was selected for all subsequent experiments.

### 2.5 The effect of elastin depletion on fascicle and IFM mechanical properties

Approximately 45 fascicles and 45 IFM samples (approx. 40 mm in length) were dissected from the mid-metacarpal region of each SDFT quarter as described previously [24], [25], and divided into 3 groups: fresh, control and 2 U/ml elastase (*n* = 15 samples per group from each biological replicate). Samples in the “fresh” group were maintained for up to 16 h in wet tissue paper at 4 °C until testing, whilst samples in the elastase and control groups were incubated in buffer solution under gentle agitation, with and without the inclusion of elastase, for 16 h at room temperature prior to the start of testing. After incubation, samples were rinsed twice in PBS then maintained on tissue paper dampened with PBS, ready for testing. For each sample, fascicle diameter was first measured using a non-contact laser micrometre [24], [25], assuming a circular shape to calculate cross section area (CSA). The mechanical properties were then determined using an electrodynamic testing machine (Instron ElectroPuls 1000) with a 250 N load cell, as previously described [5]. Briefly, fascicles were secured in pneumatic grips (grip to grip distance: 20 mm; gripping pressure 4 bar) and pre-loaded to 0.1 N, which represents approximately 2% of fascicle typical failure load. Fascicles were then preconditioned with 10 sinusoidal loading cycles (sufficient to stabilise the loading response [5]) between 0 and 3% strain (approx. 25% of failure strain; frequency: 1 Hz), immediately followed by a pull to failure test at a strain rate of 5% per second.

IFM samples were secured in the same manner and pre-loaded to the smallest positive load value that could be detected (approximately 0.02 N; equivalent to approximately 1.5% failure load). Samples were pre-conditioned with 10 loading cycles between 0 and 0.5 mm of extension (approx. 25% of failure extension; sine wave; frequency: 1 Hz), and pulled apart to failure at a speed of 1 mm/s.

All samples were kept hydrated with a mist of PBS during testing. Force and displacement data for all samples were continuously recorded at 100 Hz during both preconditioning and pull to failure, and where appropriate, engineering stress and strain were calculated using the CSA and effective gauge length, respectively. Whilst recent studies indicate that IFM thickness is roughly 20 μm, this is highly variable within and between tendons [26], [27], therefore data were maintained as force and extension to ensure consistency. Force data were smoothed, prior to any calculations, using a 9-point moving average filter, to remove noise [24]. Displacement at which the initial pre-load was reached was taken as the start point for the test to failure in both fascicles and IFM samples. Maximum stiffness (for IFM samples) or modulus (for fascicles) was identified by taking continuous tangent calculations across every 9 data points of the respective pull to failure curve, then identifying the peak value. A piecewise linear function was applied to the stress-strain data from the fascicle tests via a custom R script (www.R-project.org) to calculate the transition strain and toe modulus for each fascicle, similar to the bi-linear model approach described previously [28], [29]. Hysteresis was calculated as the difference in area under the loading and unloading curves from the first to tenth preconditioning cycles. After analysis, any fascicle or IFM sample in which failure properties or maximum modulus/stiffness were more than 2.5 times above or below the standard deviation of the mean, were excluded.

### 2.6 The effect of elastin depletion on IFM fatigue properties

To investigate the effects of elastase treatment on IFM fatigue properties, the fatigue properties of fresh, control and elastase treated IFM samples (*n* = 15 samples per group from each biological replicate) were explored, using a mechanical testing machine (Electroforce 5500, TA instruments, Delaware, USA), housed within a cell culture incubator (37 °C, 20% O_2_, 5% CO_2_), with a 22 N load cell. Samples were dissected as previously described, then secured in a custom designed chamber (grip to grip distance of 10 mm) which was filled with PBS to prevent samples from drying out. IFM samples were pre-loaded to 0.02 N and the load for creep tests was determined by first carrying out a single displacement-controlled cycle to 1 mm extension, and selecting the peak load reached. We have previously shown this to equate to approximately 50% of the predicted failure extension and to give the most consistent conditions for a controlled creep test [30]. The identified load was applied cyclically to the samples at a frequency of 1 Hz until sample failure. The average load applied was 0.7 ± 0.5 N, which equates to approximately 30% of IFM failure load. Maximum and minimum displacement data were continuously recorded at 100 Hz throughout the tests.

From the resulting creep curves, the number of cycles to failure, the creep between cycles 1 and 10 (mm), and the secondary creep rate (mm/cycle) were calculated. Data were compared between treatments and age groups. Samples in which no secondary creep was evident (immediate failure) were assumed to be damaged and rejected from the data set.

### 2.7 The effect of elastin depletion on IFM recovery

**Fig. 1:**
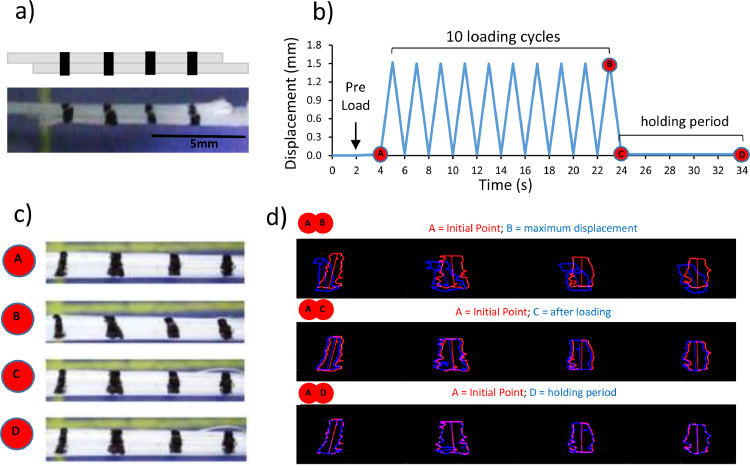
**Schematic showing IFM recovery testing protocol.** To test the ability of the IFM to recover, the opposite ends of each fascicle were removed, so that only 10 mm of intact IFM was left connecting the fascicles. Four lines were drawn across the two fascicles in the central test region to track local displacements; samples shown schematically and pictorially, where samples are pictured on a blue cutting board (a). A time-displacement graph pictorially represents the test protocol and times at which images were analysed. Four specific test points were identified; A: initial point, B: maximum displacement, C: immediately after removal of load and D: after a 10 second recovery period (b). Still frames of the sample were extracted from the video at each selected time point (c). Tracking algorithms were adopted to investigate the displacement of the lines, and thus fascicles, during each test (d).

A custom designed tensile straining rig was used to investigate the ability of the IFM to recover from loading in fresh, control and elastase treated samples (*n* = 2 samples per group from each biological replicate). IFM samples were prepared with a 10 mm test region, by cutting opposing ends from two adherent fascicles as described for the failure and fatigue tests, and secured in the rig at a 15 mm grip-to-grip distance, such that a single intact fascicle was held at each end, with the 10 mm IFM testing region in the middle (Fig. 1a). Once samples were secured, four equally spaced lines were manually drawn across the 10 mm IFM testing region using a permanent marker pen. Grips were then secured into the rig and the sample immersed in PBS. A Canon EOS 700D camera with a Sigma 105 mm F2.8 EX DG MACRO OS lens, fixed to a tripod was placed directly above the sample at a consistent height and location, to allow visualisation of sample movement. Controlled by linear actuators, the grips were slowly moved apart (at 0.05 mm/s), applying small increments of displacement, whilst visually monitoring the sample until it lifted slightly off the base of the rig, which provided a consistent start point for tests [31].

Samples were subjected to 10 loading cycles at 0.5 Hz between 0 mm and 1.5 mm (which corresponds to approximately 75% of IFM predicted failure extension), followed by a ten seconds hold period at 0 mm displacement to allow for any IFM recovery (Fig. 1b). Video footage was recorded at a rate of 10 frames per second throughout the test. At the end of the test, samples were pulled to failure at a rate of 1 mm/s, to ensure samples had been prepared correctly with both fascicles fully cut and no intact fibres traversing the test region. Samples subsequently shown to have intact fibres traversing the testing region were excluded from the data set.

### 2.8 IFM recovery - data analysis

The frames relating to specific time points during the test, designated A, B, C and D (Fig. 1b) were selected for further analysis, measuring the angular deviation of lines during the test (Fig. 1c). To briefly describe the process, Fiji (ImageJ) was used to draw a region of interest (ROI) around the marker lines, allowing the relevant image region to be cropped (Fig. 1c). The cropped images were first smoothed in MATLAB using a Gaussian filter and then thresholded, using the same parameters for all images (sensitivity 0.1; marker margin 10, chosen from preliminary experiments). Each marker line in the cropped image, was divided into a stack of horizontal lines, and for each one, the midpoint was found, and an interpolated line drawn through the midpoints (to give a line of single pixel thickness tracing the middle of the marker). The angular orientation of each line constructed in this way, was calculated relative to its orientation in the reference image (initial point - “A “), and then the average angular deviation across all four lines calculated and reported at each time point. From these data, percentage recovery after loading (comparing point C and B) and the total recovery (comparing point D and B) were also calculated.

In order to determine the potential error in measurements, the impact of shifting one end of the interpolated line by 1 pixel was investigated, demonstrating that this would affect the calculated angle by approximately 0.2°. Thus, all angular deviation values lower than 0.2° were excluded from the data set.

### 2.9 Statistical analysis

All statistical analyses were carried out using Minitab 17. Data were tested for normality using the Anderson – Darling test. If normally distributed, a two-way ANOVA, followed by Tukey post-hoc analysis was used to evaluate differences between treatments and age groups. Treatment and horse age were used as factors for the ANOVA, and each donor was nested with horse age to account for the use of multiple samples from individual donors. Data that did not follow a normal distribution were first transformed using a Box Cox transformation, and if still not normally distributed, a nonparametric Mann–Whitney test was used. Results were considered statistically significant if *p*<0.05. Data in bar graphs are displayed as mean ± standard deviation. Box plots graphs show all data points.

## 3 Results

### 3.1 Elastin depletion validation

**Fig. 2:**
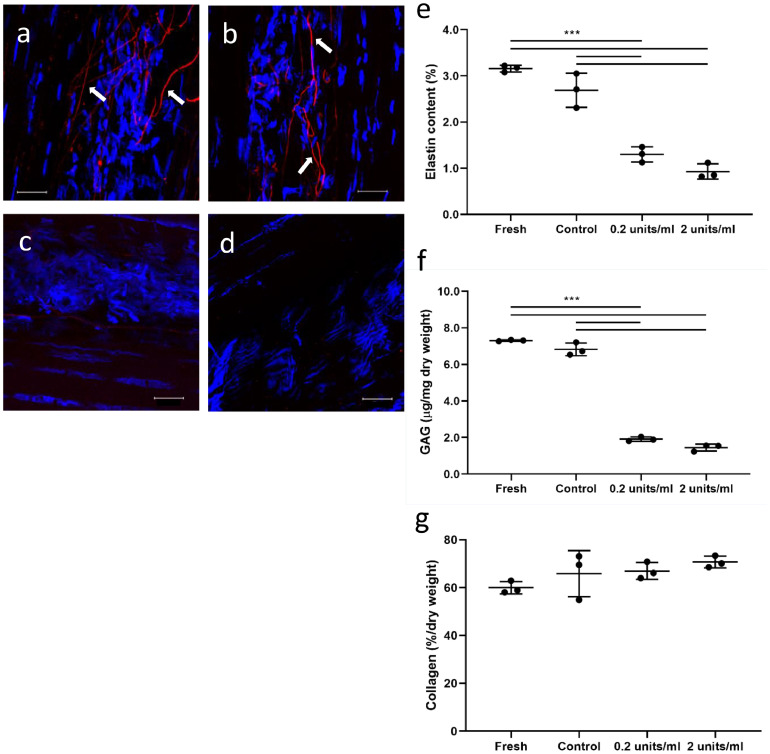
**Validation of elastase treatment.** (a-d) Representative confocal images showing tendon explants immunolabelled for elastin (red) and cell nuclei (blue): fresh (a), after incubation in control buffer (b), in 0.2 U/ml elastase solution (c) and 2 U/ml elastase solution (d). Scale bar: 20 μm. Visible elastin fibres are noted with arrows. Quantitative investigation of tendon matrix composition compares elastin (e), GAG (f) and collagen (g) content for fresh, control and elastase treated samples. Significant differences between treatments are identified by: *** *p*<0.001 (normally distributed data – ANOVA). Data are displayed as mean ± standard deviation.

Immunolabelling confirmed the presence of elastin, predominantly localised to the IFM region of both fresh (Fig. 2a) and control (Fig. 2b) samples. Very little elastin remained in the samples treated with 0.2 U/ml elastase (Fig. 2c) and no elastin was seen in the 2 U/ml elastase treated samples (Fig. 2d). Results from biochemical analysis showed an elastin reduction of over 70% when samples were incubated in a 2 U/ml elastase solution (Fig. 2e). The low amount of elastin remaining might be in the form of elastin fragments trapped in the tissue, which were either too small to visualise with immunohistochemistry or digested in such a manner that the elastin antibody no longer recognised them.

Elastase treatment also resulted in a significant decrease in GAG content, particularly in a 2 U/ml elastase solution (>80%) (Fig. 2f). As such a reduction in GAG content may have impacted the study findings, an additional investigation was carried out to determine the impact of GAG removal on IFM and fascicle mechanics, using chondroitinase ABC treatment for GAG removal. Full methods and results of the fascicle and IFM pull to failure studies after chondroitinase treatment are provided in the supplementary information. No differences in the mechanical properties of fascicles or IFM were evident in any of the GAG depleted samples.

Collagen content was unaffected by elastase treatment (Fig. 2g).

Based on these results, 2 U/ml elastase was used in all subsequent experiments.

### 3.2 Fascicle & ifm failure properties

Fascicle and IFM failure properties are shown in Table 1. Fascicle failure tests showed no significant differences in any mechanical parameters between treatment groups, nor any significant differences with ageing. By contrast, elastase treatment led to a significant reduction in IFM failure load and maximum stiffness in both young and old groups, and an overall increase in IFM hysteresis in elastase treated samples (Table 1). No significant differences between fresh and control samples were found in any of the parameters assessed, indicating that differences resulted from elastase treatment and not incubation. The majority of variables were unaffected by ageing, with the exception of failure extension and initial hysteresis, which showed small but significant decreases with ageing in elastase treated IFM samples.

**Table 1:** **Fascicle & IFM Failure Properties in fresh, control, and elastase treated samples from young and old horses.***n* = 5/age group; total samples tested: 15/treatment/tendon. Data are shown as mean ± SD. Significant differences are flagged with: *p < 0.05, **p < 0.01 and ***p < 0.001. CE indicates differences between control and elastase treated groups; FE, differences between fresh and elastase groups.

Fascicles	Young SDFT				Old SDFT				
	Fresh (F)	Control	Elastase	Within Age	Fresh (F)	Control	Elastase	Within Age	Between age
		(C)	(E)	group		(C)	(E)	group	groups
				comparisons				comparisons	comparisons
Diameter (mm)	0.24 ± 0.04	0.23 ± 0.04	0.23 ± 0.04		0.23 ± 0.05	0.21 ± 0.05	0.22 ± 0.05		
Failure Load (N)	2.2 ± 0.7	2.2 ± 0.9	2.1 ± 0.8		2.0 ± 1.0	1.8 ± 0.9	1.8 ± 0.8		
Failure Stress (MPa)	50 ± 14	51 ± 14	49 ± 19		47 ± 18	55 ± 25	51 ± 21		
Strain at Failure (%)	11 ± 2	12 ± 2	12 ± 2		11 ± 3	11 ± 2	11 ± 2		
Maximum Modulus (MPa)	649 ± 140	644 ± 150	630 ± 205		631 ± 156	748 ± 260	676 ± 220		
Transition strain (%)	1.4 ± 0.1	1.4 ± 0.2	1.4 ± 0.2		1.4 ± 0.2	1.3 ± 0.3	1.4 ± 0.2		
Toe Modulus (MPa)	355 ± 87	381 ± 117	359 ± 118		363 ± 109	449 ± 216	425 ± 163		
Hysteresis Cycle 1 (%)	26 ± 4	24 ± 4	26 ± 3		23 ± 5	23 ± 4	24 ± 4		
Hysteresis Cycle 10 (%)	10 ± 2	9 ± 2	9 ± 2		9 ± 2	9 ± 2	9 ± 2		

IFM Samples	Young SDFT				Old SDFT				
	Fresh (F)	Control	Elastase	Within Age	Fresh (F)	Control	Elastase	Within Age	Between age
		(C)	(E)	group		(C)	(E)	group	groups
				comparisons				comparisons	comparisons

Failure Load (N)	1.6 ± 0.7	1.5 ± 0.6	1.0 ± 0.4	***FE; ***CE	1.6 ± 0.6	1.5 ± 0.6	0.9 ± 0.4	***FE; ***CE	
Failure Extension (mm)	2.0 ± 0.6	2.1 ± 0.6	1.9 ± 0.6		1.7 ± 0.5	1.8 ± 0.4	1.5 ± 0.4		*C; **E
Maximum Stiffness (N/mm)	1.3 ± 0.4	1.2 ± 0.3	0.9 ± 0.2	***FE; **CE	1.5 ± 0.4	1.3 ± 0.3	0.9 ± 0.2	***FE; ***CE	
Hysteresis Cycle 1 (%)	32 ± 6	32 ± 4	39 ± 7	**FE; **CE	30 ± 6	33 ± 7	35 ± 7	***FE	*E
Hysteresis Cycle 10 (%)	13 ± 3	13 ± 2	17 ± 7	***FE; **CE	14 ± 5	16 ± 7	18 ± 13		

### 3.3 IFM fatigue properties

**Fig. 3:**
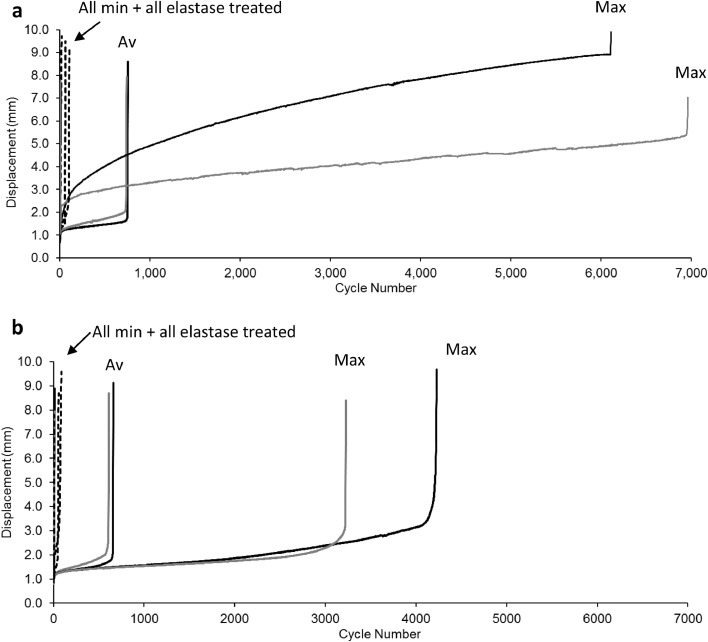
**Creep curves of young SDFT IFM (a) and old SDFT IFM (b), highlighting the variability between samples by plotting the maximum (Max) and minimum (Min) curves in each group, alongside the average (*Av*).** Fresh samples are shown in black, control samples in grey, elastase treated with a dotted line. Labelling is not used for elastase treated samples as it evident all elastase treated samples failed at less than 100 cycles.

**Fig. 4:**
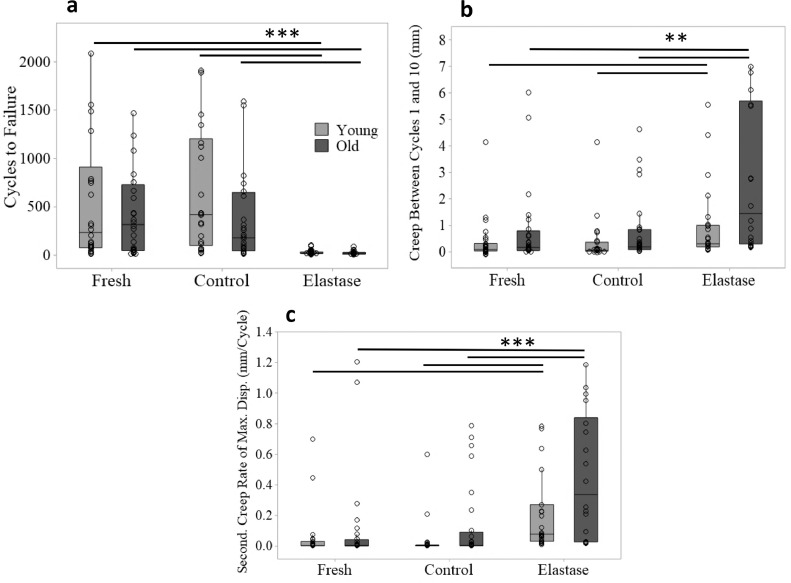
**Effect of elastin depletion on IFM fatigue properties in young and old samples.** Data compares cycles to failure (a), creep between cycles 1 and 10 (b) and secondary creep rate of maximum displacement (c) for young and old SDFT IFM samples from fresh, control and elastase treated groups (*n* = 5/age group; total samples tested: 15/treatment/tendon). Significant differences are flagged with: **p<0.01 and ***p<0.001 (Cycles to Failure: normally distributed – ANOVA, all remaining IFM fatigue data not normally distributed – Mann-Whitney test).

Representative creep curves for young and old SDFT IFM samples are shown in Fig. 3, whilst all SDFT IFM fatigue data are summarised in Fig. 4a-c.

Data show a significant reduction in the number of cycles to failure in elastase treated samples compared to both fresh and control groups, despite the large variability within treatment groups (Figs. 3 and 4a). Results also show that elastase treatment led to a significant increase in creep between cycles 1 and 10 (Fig. 4b) and secondary creep rate, compared to both fresh and control groups (Fig. 4c). The response to elastase treatment did not differ significantly between young and old samples, however there was notably greater variability in aged samples, potentially masking any age-related changes (Fig. 4).

### 3.4 IFM recovery properties

**Fig. 5:**
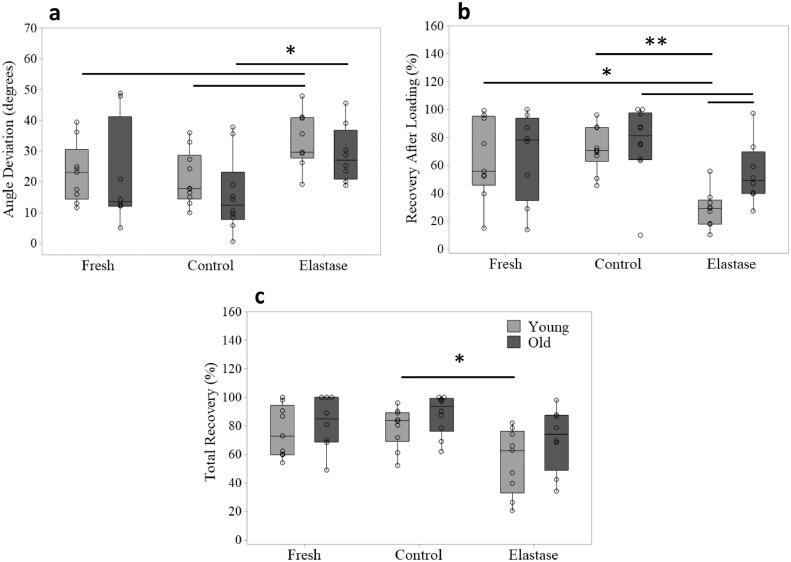
**Effect of elastin depletion on IFM loading response and recovery.** Loading and recovery were visually monitored by tracking markers across the IFM samples. Angle deviation (degrees) of lines was first determined under the application of 75% of the predicted failure extension (a). Load was removed, and then recovery of lines relative to their start point was measured immediately after loading (b) and 10 s after the removal of load (c). Graphs compare young and old SDFT IFM samples in the fresh, control and elastase treated groups (*n* = 5/age group; total samples tested: 2/treatment/tendon). Significant differences are flagged with: *p<0.05 and **p<0.01 (data non-normally distributed – Mann-Whitney test). For more details regarding loading protocol refer to Fig. 1.

The analysis of IFM recovery images is shown in Fig 5. Data demonstrated a significant increase in the angular deviation at the peak of applied load in the elastase group compared to both fresh and control groups (Fig. 5a), indicating increased fascicle sliding after elastase treatment. On removal of load, the percentage recovery (Fig. 5b) was significantly lower in the elastase group compared to either of the other groups, and remained significantly reduced even after a hold period (Fig. 5c).

In aged samples, overall trends were similar, but it was notable that immediate recovery after loading was significantly better in the aged than young elastase treatment group (Fig. 5b). There were no significant differences between fresh and control groups in any of the calculated variables.

## 4 Discussion

This is the first study to elucidate the regional-specific influence of elastin on energy storing tendon mechanics, exploring the impact of elastin depletion on the quasi-static and viscoelastic behaviour of the IFM and fascicles in the context of ageing. Data support the hypothesis that elastin depletion exclusively impacts IFM mechanical properties, and also illustrate a consistent trend towards a more pronounced effect in young samples. All elastase-treated IFM samples consistently show a significantly reduced ability to withstand applied load, resist fatigue loading, and recover after loading.

This study specifically selected the equine model, owing to its relevance as an energy storing tendon with similar structure, disease pathology and epidemiology to that seen in human energy storing tendons [17]. This is essential for the current study, in which we hypothesise the importance of specific regions of tendon (the IFM), the structure and composition of which is similar between species [32].

The need to adopt a large animal model requires the use of enzymatic digestion approaches to carry out structure-function studies, which is associated with a number of limitations [18], [33]. Enzymatic digestion studies necessitate incubation within a buffer solution, with previous work demonstrating that the buffer solution alone can cause swelling and likely impact tendon mechanics [34], [35]. Appropriate buffer-only controls were utilised to help differentiate the impact of buffer solutions and the enzymes, and these demonstrated that mechanical changes were specific to the digest group. However, it is also well acknowledged that enzyme efficacy and specificity must be considered for targeted digestion assays. Use of proteinase-free enzyme preparations and inclusion of a trypsin inhibitor to prevent collagen degradation [21] provided the ability to remove 70% of elastin from tendon without affecting tendon collagen content. However, elastase treatment did cause significant GAG depletion (>80%).

Some previous studies have reported a similar reduction of GAG content in other tissues exposed to elastase, likely occurring because GAG is less tightly bound within the extracellular matrix than other matrix components, and thus easily released by any disruption [18], [36], [37]. Proteoglycans interact with collagens and elastin microfibrils via their GAG sidechains, where they are believed to contribute to microfibril integration into the extracellular matrix [38]. Therefore, we performed additional experiments to determine if GAG removal alone affected IFM and fascicle mechanical properties. The results demonstrated no discernible effects of 90% GAG removal on IFM or fascicle mechanical behaviour (Supplementary Information). These findings support previous studies showing that tendon mechanical properties are not significantly affected by GAG digestion [39], [40] and provide confidence that any changes in tissue mechanics originate from loss of elastin and not GAGs.

Data demonstrate no changes to fascicle quasi-static or viscoelastic properties post-elastase treatment. With the majority of elastin in tendon localising to the IFM, and fascicles comprising more than 90% collagen, these findings are perhaps not surprising [9]. By contrast, after elastase treatment, IFM failure load and stiffness were both significantly reduced compared to both fresh and control groups, and data demonstrated a significant increase in hysteresis in elastin depleted IFM samples, and such a significant increase in the levels of primary and secondary creep, and reduction in fatigue resistance, that most samples failed immediately. Unexpectedly, ageing had little effect on IFM response to elastase treatment, with only small decreases in failure extension and initial hysteresis evident when compared to young elastase treated samples. Indeed, we did not identify any decrease in fatigue resistance with ageing in the control samples. This is in contrast to previous studies which identified a significant decrease in IFM fatigue resistance with ageing [11]. The reasons for this remain unclear, but may be a result of variability in the age-associated changes in elastin. In our previous work, we have observed a reduction in elastin content, in the equine SDFT with ageing, with the remaining elastin becoming disorganised [9]. The causes of this remain unclear, but may be due to fatigue loading or enzyme-mediated degradation, as has been observed in arterial elastin [41], [42].

Interestingly, no changes were observed in the failure extension of SDFT IFM samples after elastase treatment. With increased hysteresis evident in these samples, this finding was surprising, but we speculate that it arises from the protocol adopted after the preconditioning. Sample extension was normalised after the 10 preconditioning cycles and prior to the pull to failure as per previous studies [5], meaning any irrecoverable extension that occurred during the preconditioning cycles was not included in the reported values. With our results also demonstrating poor recoverability of the IFM in elastase treated samples, and previous studies showing increased lengthening of elastase treated ligaments under pre-stress [21] it seems likely that the elastase treated IFM extended notably during those initial cycles, as the IFM was less able to sustain applied load.

Taken together, our data indicate that elastase treatment specifically impacts the IFM region of tendon without affecting fascicle mechanical behaviour, and elastin depletion leads to the IFM becoming less able to withstand load, with reduced fatigue resistance. Few previous studies have investigated the effect of elastase treatment on fascicles specifically, although it has been shown that elastin depletion of rat tail tendon (RTT) fascicles resulted in a reduction in failure stress and strain [18]. It is unclear why these findings are in contrast to those we report here, although significantly longer digestion times and higher incubation temperatures were used by Grant et al. [18]. Perhaps, the inherent function of the RTT versus the SDFT could also be a reason. The energy storing capacity of the SDFT when compared to the RTT could result in differences in elastin distribution between these two tissues. Additionally, different animals have different activity requirements, which might also be a reason for the distinct results. No previous studies have assessed the influence of elastin removal on IFM mechanics specifically. A number of studies have considered the impact of elastin depletion on whole tendon or ligament mechanics, demonstrating a reduction in tissue stiffness and/or failure stress post-elastase digestion [21], [43]. However, the effects of elastase on whole tendon viscoelastic properties remain unclear, with some studies reporting increased hysteresis [43], whilst others show no alterations [12], [21]. These differences are likely a combination of inherent differences between tendon and ligament response to elastase treatment, as well as differences in elastase treatment and mechanical testing protocols used. To the authors’ knowledge, no previous studies have directly investigated the effect of elastin depletion on tissue fatigue properties.

IFM testing in the current study was carried out in shear, pulling the opposing ends of adjacent fascicles, and demonstrating that the IFM possessed reduced ability to resist shear stresses after elastase treatment. Interestingly, whilst no comparable IFM mechanical tests exist, there are a number of studies investigating the effect of elastase treatment on either the transverse or shear mechanical properties of tendon, which would likely augment the influence of the IFM on resulting data. These studies report decreased shear and transverse stresses in both tendon and ligament after elastin depletion [20], [44]. Taken together, these results support the hypothesis that elastin provides a mechanical link between fascicles, providing the capacity to resist shearing.

Direct optical measurement of local strains in the IFM during loading and recovery provided greater insight into the mechanical behaviour reported. Data revealed a significant increase in the angular deviation during loading in the elastase group compared to both fresh and control groups, suggesting that elastase treated IFM samples stretched and sheared more than fresh and control samples, when subjected to similar displacements. The additional IFM extensibility in elastase treated samples appears contradictory to the earlier reported lack of change in IFM failure extension. However, IFM failure extension data only reported extension in the final pull to failure test, and not that occurring during preconditioning cycles. Angular deviation measurements will determine the sum of all extension from the beginning of the first loading cycle, and imaging demonstrated that the IFM responded immediately to applied load with irrecoverable extension and shear.

Optical imaging also enabled a closer investigation of recovery behaviour, showing a significant decrease in the percentage recovery of IFM sliding after load was removed in the elastase group compared to all other treatment groups. Indeed, while recovery in fresh and control samples was close to 70%, this was reduced to 30% in elastase treated samples from young donors. Providing a hold period in the unloaded state for further recovery resulted in little further change in fresh and control samples, probably because most of the recovery had already been observed immediately after load was removed. By contrast, it was interesting to note that elastase treated samples from young donors showed continuing recovery during the holding period. However, the total recovery of these samples was still significantly lower than that seen in control groups.

Unexpectedly, immediate recovery from loading in elastase treated samples was significantly better in old than young IFM samples. It has previously been established that tendon elastin content decreases with ageing [9], from which it can be inferred that elastase will have less impact on aged samples. However, these findings do suggest that age-associated changes in IFM structure may lead to collagens, proteoglycans and/or other components of elastic fibres, such as fibrillin and fibulin, contributing to IFM recoil in older tendons.

Whilst we note that samples were frozen, in fresh tissue it is possible that a viable cell population may impact mechanical behaviour. Taken together, data imply that elastase treatment results in increased IFM sliding and reduced ability of the IFM to withstand loading or recoil after the load is removed, all contributing to the observed reduction in fatigue resistance.

## 5 Conclusions

These findings are of crucial importance to structure-function studies, allowing a new level of insight into the hierarchical mechanics of tendon and highlighting the important contribution of elastin to tendon mechanical properties. Data demonstrate that maintenance of a functional elastin network within the IFM is critical to maintain IFM and, consequently, tendon integrity.

## Funding Sources

This study was funded by the BBSRC (BB/K008412/1). MG was funded by a QMUL Bonfield PhD scholarship. The funders had no role in study design, collection, analysis and interpretation of data, writing of the report; and in the decision to submit the article for publication.

## Data availability statement

All data are available from the authors on reasonable request.

## Declaration of Competing Interest

The authors declare that they have no known competing financial interests or personal relationships that could have appeared to influence the work reported in this paper.

## Appendix Supplementary materials

Image, application 1
